# What Will Probably Be the Dental Educational Standard for the Coming Decade?1Read in the Section on Stomatology of the American Medical Association, at the fifty-sixth annual session, July, 1905. From the Journal of the American Medical Association.

**Published:** 1905-09

**Authors:** Charles C. Chittenden

**Affiliations:** Madison, Wis.


### WHAT WILL PROBABLY BE THE DENTAL EDUCATIONAL STANDARD FOR THE COMING DECADE?[1]

BY CHARLES C. CHITTENDEN, D.D.S., MADISON, WIS.

At the last meeting of this Section, at Atlantic City, in 1904, a symposium on dental educational standards was read, which was exhaustive and thorough in its character. In a paper presented at that symposium I had the honor to report chronologically the various facts as they had transpired, during the current year, concerning the attitude of the individual dental schools and the National Association of Dental Faculties toward the newly inaugurated four years' college course.

It had become apparent that a large number of the smaller and financially hampered schools were determined on returning to the three years' course.

This Section, at that time, in its discussions, expressed, in no uncertain terms, its entire disapproval of any such retrograde action.

Immediately following the meeting of this Section came the annual meeting of the National Association of Dental Faculties at Washington, D. C., where the whole subject was gone over and discussed very exhaustively, with the final result that, by a close vote of twenty-four to twenty-one colleges, the four years' course was upheld. This occurred in the early part of June. Almost at once following the adjournment the *ad interim* committee began to receive the resignations from membership in the National Association of Dental Faculties of a number of schools which were opposed to the decision at Washington to continue the four years' course.

---

[1] Read in the Section on Stomatology of the American Medical Association, at the fifty-sixth annual session, July, 1905. From the Journal of the American Medical Association.

These resignations created such a panic in the ranks of the colleges that the *ad interim* committee was finally induced to call a special meeting of the National Association of Dental Faculties to be held at St. Louis, July 18, 1904, whose sole purpose should be a reconsideration of the final decision made at Washington, the month previous, to continue the four years' course.

At this special meeting there were, of the fifty-one colleges in membership, twenty-eight represented. By a vote of 26 to 2 (being by a majority of one of the total membership) the four years' course was revoked and a three years' course of thirty weeks in each year was adopted in its place. The public announcement of this distinctly retrograde step (taken by a bare majority of one of the membership of the college association) came as a distinct shock to the rank and file of the profession. There was no semblance of an off-set in the way of increased standard requirements for admission to the course to, in some measure, soften the baldness of the action. The outside world was simply made suddenly aware that the National Association of Dental Faculties, without waiting to graduate a single class or man under the vaunted higher educational system which that body had spent at least three years in elaborating, and had then declared to the world as absolutely necessary to properly fit the student for the dental degree, had, without explanation, struck its colors and surrendered. The one saving phase of the whole transaction was the fact that practically one-half of our colleges had been opposed to the change and had only acquiesced in it "to save the Faculties Association."

The examiners of the United States had been loyally standing behind and ready to protect the schools in their higher standards in every way. To them this bold retrogressive step called for immediate action. A blow had been struck, for commercial reasons only, at the established standards, and struck, too, by our National Association of Dental Faculties! All the schools of the better class had been obliged to yield to the inevitable and lower their standards—all save one, whose noble independence but makes the situation appear the more pathetic.

The annual meeting of the National Association of Dental Examiners was held late in August, 1904, at St. Louis. It was apparent, from the first news of the "retrogression," that something must be done to retrieve the situation before the world, and the examiners rose at that meeting to the occasion. The following report from the Committee on Colleges, preceded by a careful *résumé* of the entire situation, was unanimously adopted:

Your committee would therefore recommend that this association establish at once, to go into operation not later than the opening of the school year of 1905–6, the educational requirements, for admission to the dental college course, of graduation from an accredited high school or its full equivalent, all examinations of credentials and equivalents to be placed in the hands of an acceptable appointee of the State Superintendent of Public Instruction where not otherwise provided for by law.

In view of the present disturbed and unsettled conditions existing in dental educational circles, and with a belief in avoiding all unnecessary disturbances of standards at this time, your committee would further recommend that no change be made at this time in the present requirements of this association of not less than twenty-eight calendar months of college attendance for graduation.

By what had occurred the trust of the examiners in the ability of the college association to maintain good faith under certain contingencies was so badly shaken that all standing resolutions which in any way interfered were rescinded, and the Committee on Colleges was instructed to prepare a new list of recommended colleges, based on the acceptance by the individual schools of the standards declared in the above report. The work was to be done independently of the National Association of Dental Faculties.

After having spent many months in correspondence and careful conferences with many of our ablest teachers and scientists, the Committee on Colleges issued the following letter to the deans of this country, February 14, 1905:

In consideration of the conflicting views as to dental educational standards which have existed for some time, the National Association of Dental Examiners at its annual meeting held at St. Louis, August, 1904, deemed it expedient, and necessary for the upholding of such schools as sought to maintain the standards already published to the world as the minimum that should obtain, to declare what educational standards should be required by the State Boards of Examiners as a criterion of reputability of the schools seeking recognition of their output.

This *ad interim* committee, which is also the committee on colleges, was instructed to inform all schools of the action taken, and directed to prepare a recommended list of colleges on the basis of the standards established at that meeting.

Feeling fully the gravity of the duty imposed, this committee has expended much effort in striving to arrive at a basis of fairness to all interests concerned, in carrying out its general instructions. The chief requirement established at St. Louis was that of "graduation from an accredited high school or its full equivalent" for admission to the classes of 1905–6.

In several schools and university departments this requirement is already in actual operation, and our committee finds a considerable number of other schools desiring to maintain it. All these, of course, will be placed on the recommended list. There are, however, other schools whose deans assert that to enforce at once this advance requirement would work a serious financial injury to their institutions.

The question of what would constitute a proper length of course for graduation from a dental college has always been left by the examiners to the colleges themselves, except that, after a school has announced to the public a certain course as necessary to properly fit a student for graduation, if it for private or financial reasons deliberately lowers its requirements in any particular, the question of good faith and reputability of that school becomes at once a matter for adjudication by every board in the country.

We, therefore, acting on authority of, and, *ad interim*, representing the National Association of Dental Examiners, which is the advisory body of the various State boards in their official acts, respectfully request that you authorize the Committee on Colleges to place your school on the recommended list of colleges by the acceptance of the following educational requirements for students, viz.:

For matriculation or registration, "graduation from an accredited high school or its full equivalent, all examination of credentials and equivalents to be placed in the hands of an acceptable appointee of the State Superintendent of Public Instruction where not otherwise provided for by law," said requirements to be inaugurated not later than the beginning of the school year of 1906–7; and a college course for graduation optional with you of either four years of seven months each or three years of nine months each, this course requirement to be inaugurated the present year, *1905*.

It is to be expected that schools maintaining these standards will be protected in so doing by the several boards composing the National Association of Dental Examiners.

It is the intention of this committee to prepare and to publish the recommended list of colleges not later than April 1 next, in order to give all schools the earliest opportunity to announce these standards to the public. Therefore information as to your decision is desired as early as possible.

Very respectfully yours,

COMMITTEE ON COLLEGES.

The responses have been quite general and, on the whole, unexpectedly satisfactory. It developed that a large number of schools were only too desirous for the establishment of an educational requirement at once reasonable and at the same time so sufficiently advanced as to not only retrieve the unfortunate back step of 1904, but also to place their schools on a permanent working basis so advanced as not to be liable to material change for several years to come. As one dean expressed it, "This higher standard places us in a position to go ahead with our business, and we will not have to change again unless the Faculties' Association goes one better. It has been this uncertainty as to what was coming or what we were going to do next that had troubled me most. Now I feel as though we had something definite before us."

And thus it has happened that, up to this date, the acceptances have far outnumbered the refusals and the new college list is still growing.

The *rationale* of the matter being that while the National Association of Dental Faculties may and has set minimum bounds of requirement for its members, it can not and never will undertake to prohibit any or all of its membership from placing their individual requirements as much higher as they may see fit. The only chance taken by the individual school in so doing is as to the ability of its product to compete successfully in the market with that of the schools retaining the lower standards.

The question, therefore, resolves itself to this: Will the examiners, with the power of law behind them, keep faith and redeem the pledges made by their authority and in their name by their chosen representatives, *i.e.*, stand by and judicially maintain the advanced educational requirements established by the National Association of Dental Examiners at St. Louis in 1904?

The faith manifested by more than a score of our foremost schools and universities in the integrity and honor of the examiners in this respect would seem to be a harbinger of a new order of things which will be, at least, paramount to commercial success in the conducting of educational institutions.

If the hopes herein foreshadowed shall become realities, a new impetus will be given to dental education, a better class of minds will be attracted to our schools, and for many years to come there will be no further disturbance in preliminary educational requirements for entering our dental colleges.

